# Nephronophthisis-Pathobiology and Molecular Pathogenesis of a Rare Kidney Genetic Disease

**DOI:** 10.3390/genes12111762

**Published:** 2021-11-05

**Authors:** Shabarni Gupta, Justyna E. Ozimek-Kulik, Jacqueline Kathleen Phillips

**Affiliations:** 1 Macquarie Medical School, Faculty of Medicine, Health and Human Sciences, Macquarie University, Sydney, NSW 2109, Australia; justyna.ozimek-kulik@hdr.mq.edu.au (J.E.O.-K.); jacqueline.phillips@mq.edu.au (J.K.P.); 2 School of Women’s and Children’s Health, University of New South Wales, Sydney, NSW 2031, Australia; 3 Department of Paediatric Nephrology, Sydney Children’s Hospital Network, Children’s Hospital at Westmead, Sydney, NSW 2145, Australia

**Keywords:** nephronophthisis, kidney, cyst, polycystic kidney disease, nephrocystin, polycystin, cilia, chronic kidney disease, intraflagellar transport

## Abstract

The exponential rise in our understanding of the aetiology and pathophysiology of genetic cystic kidney diseases can be attributed to the identification of cystogenic genes over the last three decades. The foundation of this was laid by positional cloning strategies which gradually shifted towards next-generation sequencing (NGS) based screenings. This shift has enabled the discovery of novel cystogenic genes at an accelerated pace unlike ever before and, most notably, the past decade has seen the largest increase in identification of the genes which cause nephronophthisis (NPHP). NPHP is a monogenic autosomal recessive cystic kidney disease caused by mutations in a diverse clade of over 26 identified genes and is the most common genetic cause of renal failure in children. NPHP gene types present with some common pathophysiological features alongside a diverse range of extra-renal phenotypes associated with specific syndromic presentations. This review provides a timely update on our knowledge of this disease, including epidemiology, pathophysiology, anatomical and molecular features. We delve into the diversity of the NPHP causing genes and discuss known molecular mechanisms and biochemical pathways that may have possible points of intersection with polycystic kidney disease (the most studied renal cystic pathology). We delineate the pathologies arising from extra-renal complications and co-morbidities and their impact on quality of life. Finally, we discuss the current diagnostic and therapeutic modalities available for disease management, outlining possible avenues of research to improve the prognosis for NPHP patients.

## 1. Introduction

Cystic diseases of the kidney are comprised of a diverse group of acquired, hereditary, and developmental disorders where renal cysts are the common denominator driving kidney failure. Renal cysts can develop sporadically due to developmental abnormalities or be acquired because of drugs, hormone treatments, age or dialysis [1]. Cystic kidney diseases that are genetic in origin, i.e., are caused by germ-line mutations occurring in single genes (also termed monogenic), are inherited as Mendelian traits in an autosomal dominant or recessive manner [2]. Proteins encoded by these cystogenic genes are almost always localised in the primary cilium of the cell and, therefore, these cystic kidney diseases are also termed ciliopathies (disease of the cilia). Broadly, these include polycystic kidney disease (PKD), autosomal dominant tubulointerstitial kidney disease (ADTKD), nephronophthisis (NPHP) and various other NPHP related ciliopathies (NPHP-RC) [2]. Despite having some shared pathological features, the monogenic mutations causing these diseases occur within a repertoire of diverse ciliary genes, with each having distinct pathological characteristics [3]. While traditional positional cloning strategies laid the groundwork, next-generation sequencing (NGS) substantially accelerated our pace of identifying the pathogenic genes responsible for cystic kidney diseases, with over 50 new NPHP genes alone identified in the last 10 years [3]. Given this surge of findings, and that they are the most frequent genetic cause of end-stage renal disease (ESRD) in the first three decades of life, this review focuses on subjects central to understanding the pathobiology of NPHP and NPHP-RC. This includes clinical presentation, heterogeneity across the various forms, histopathology, and epidemiology. We review current progress made in the understanding of the molecular mechanisms causing NPHP and finally, diagnosis, management strategies, and unmet clinical and research needs are discussed. 

## 2. Epidemiology

NPHP is a broad group of autosomal recessive cystic kidney disease that is the most frequent genetic cause of ESRD in the first three decades of life. NPHP was first reported as a sporadic case by Smith and Graham in 1945 [4]. Following this, reports of familial disease in two large kindreds were published by Fanconi in 1951, where he coined the term “familial juvenile nephronophthisis” [5,6]. Here, “nephronophthisis”, translating to “disappearing of nephrons”, was used to describe the histopathology of affected children [5]. Until the 1980s, over 300 NPHP cases were reported worldwide across all ethnicities [7]. More recently, the incidence of NPHP is reported to be between 1 in 50,000–900,000 and shows evidence of global variance [8]. However, these numbers may be an under-representation of the true incidence due to challenges in access to molecular testing worldwide [8]. NPHP has a prevalence of 0.96 per million population above the age of 18 years in Australia [9]. Incidence is 1 in 50,000 births in Canada [10] and 1 in 61,800 in Finland [11], while 9 cases per 8.3 million people were reported in the United States [12]. Based on the onset of ESRD, NPHP is classified into three clinical forms: infantile-, juvenile- and adolescent-onset, with a median age of ESRD at 1, 12 and 15 years, respectively. NPHP does not have a gender predisposition and accounts for 10–25% and ~5% of all children in ESRD in Europe and North America, respectively [13]. Bollee et al. [14], Hoefelle et al. [15], and Hudson et al. [16] showed new diagnosis of NPHP in adults with chronic kidney disease (CKD), despite the diseases likely being present from childhood.

## 3. Pathophysiology and Clinical Findings

**Figure 1:**
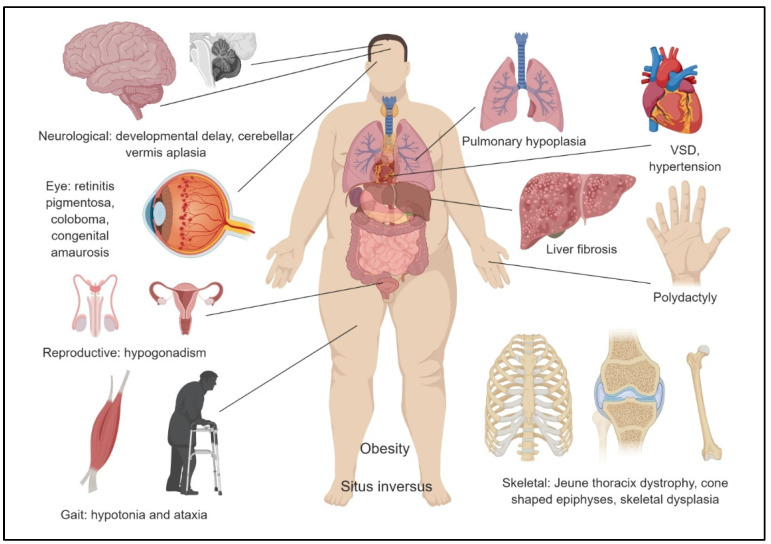
Multi-organ involvement in Nephronophthisis (NPHP) presentation: Schematic showcasing the phenotypic presentations observed in the various forms of NPHP affecting extra-renal sites. Ventricular septal defect (VSD). (Created using BioRender.com).

In being a ciliopathy, primary cilium dysfunction reflects in the pathophysiology of the various forms of NPHP as well as NPHP-RCs. The primary cilium is an antenna-like organelle that plays a pivotal role in embryonic development and laterality determination. Primary cilia are also critical for the functioning of the kidney, eye, brain, and several other organs. Pathogenic NPHP mutations resulting in malfunction of cilia involved in critical organ specific functions, can be hypothesized to be an underpinning factor governing the diversity observed in the above-described disease manifestation. Pathophysiological features of NPHP typically include kidneys with cysts and interstitial fibrosis [17]. However, as mentioned in the previous sections, NPHP types showcase phenotypic pleiotropy where 10–20% of cases present with multiorgan involvement [18] (Figure 1). The pathophysiological features observed in organs affected in certain types of NPHP are described here.

### 3.1. Kidneys

Early transmission electron microscopy studies in PKD described cilia that were either reduced or absent, with abnormal infoldings of their membrane and reduced cellular organelle [19]. Tubular basement membrane was thickened with some degree of interstitial fibrosis [20]. Addendum of scanning electron microscopy revealed different degrees of epithelial appearances within kidneys, showing that some cells looked healthy and the others overproliferated [21]. In kidneys, mutations in NPHP genes lead to the development of cysts and fibrosis. Cystogenesis is the best described in autosomal dominant PKD (ADPKD), where all segments of the nephron are affected, though the process usually starts in the collecting duct [22]. In the process of cystogenesis, over-proliferation of cells and disruption of the spatial orientation of cells leads to dilatation of the tubules, instead of longitudinal growth [23]. In NPHP, the cysts also originate in a collecting duct, but remain connected with their originating tubule (contrary to ADPKD, where cysts separate from the original tubule) [24]. Besides proliferation and expansion of tubular epithelium, tubular basement membrane disintegrates [25], and malfunctioning tubules are unable to concentrate urine. Clinical patients present with polyuria, polydipsia, nocturia [26,27] and microalbuminuria [28].

### 3.2. Eyes

NPHP can be associated with a wide spectrum of visual impairment. It can present as late onset night blindness or severe visual impairment in early infancy, and is associated with either retinitis pigmentosa, as in Senior Løken syndrome, Leber Congenital Amaurosis [29], or retinitis pigmentosa, hypopituitarism, nephronophthisis, and skeletal dysplasia (RHYNS)syndrome [30], or it can present with coloboma of the optic nerve, as in Joubert syndrome [31]. 

### 3.3. Central Nervous System

Another system frequently involved in NPHP is the central nervous system. Some of the abnormalities described include cerebellar vermis aplasia with ataxia, as seen in Joubert syndrome [31], or brainstem malformations, including so called “molar tooth sign” [18], which is a radiologic description of the appearance of the midbrain on MRI scans, where cerebellar peduncles are enlarged due to abnormal crossing of their fibre tracts [32]. Hypoplasia of cerebellar vermis is also associated with oculomotor apraxia in Cogan’s apraxia [33]. Affected individuals have absent or defective voluntary horizontal eye movements. 

### 3.4. Liver

Liver is another organ affected in NPHP, similarly to the more common ciliopathy, autosomal recessive PKD (ARPKD). In Meckel–Gruber syndrome, liver fibrosis is present together with CNS, ophthalmic and skeletal abnormalities, as well as dysmorphic features [34], while in cerebellar vermis defect, oligophrenia, ataxia, coloboma, hepatic fibrosis (COACH) syndrome, fibrosis is severe enough to cause portal hypertension [35].

### 3.5. Musculoskeletal System

Abnormalities in the formation of the cartilage, which are characterised by cone shaped epiphyses, are found in Mainzer–Saldino syndrome, also known as Conorenal syndrome [36]. In the more common Bardet–Biedl syndrome (BBS), there is usually short stature and abnormalities of the distal limb, typically polydactyly, brachydactyly or syndactyly [37]. On the more severe spectrum, Jeune syndrome, also known as asphyxiating thoracic dystrophy syndrome, is associated with short ribs, hypoplastic phalanges, polydactyly and short limbs [38], while Sensenbrenner and RHYNS syndromes are skeletal dysplasia [30,39]. 

### 3.6. Reproductive System

Hypogonadism is found in BBS [40], and it is thought to be secondary to impaired organogenesis and sperm function but possibly also a defective hypothalamic–pituitary axis [41].

### 3.7. Cardiovascular System

Blood pressure has been a focus of more recent studies including meta-analysis of hypertension in children with PKD [42], and was thought to be secondary to upregulation in renal renin angiotensin aldosterone system (RAAS). In NPHP, blood pressure rises, usually secondary to poor kidney function, though is uncommon [43]. Situs inversus and ventricular septal defects are described in NPHP, particularly in NPHP2 [44]. 

While organ specific roles of cilia could offer a plausible explanation for the diverse pathobiology observed in NPHP, the cellular roles of proteins encoded by the various NPHP genes, alongside their function in ciliary signalling, may be another facet of studying the molecular pathobiology of this disease.

## 4. Genetics

**Figure 2:**
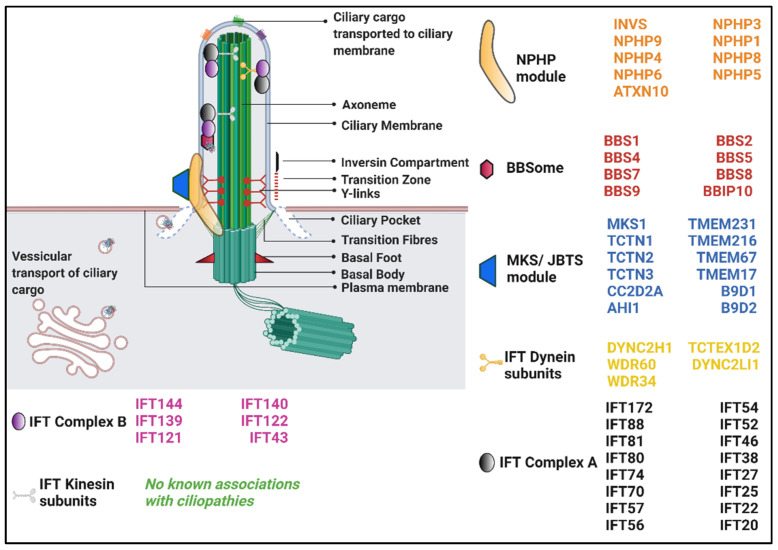
Ultrastructure of cilia and ciliopathy modules: The primary cilium is a highly compartmentalized microscopic organelle with several distinct parts (not drawn to scale). Although each compartment houses proteins belonging to different families, an aberration in their expression cause ciliopathies that can be grouped to specific modules. NPHP module includes, inversin (INVS/NPHP2). Nephrocystin 1, 3, 4, 5, 6, 8, 9 (NPHP1-9), Ataxin 10 (ATXN10); Mutations in proteins causing Bardet–Biedl syndrome (BBS) are located in the BBSome module comprising of proteins (BBS1-9, BBSome-interacting protein of 10Kda (BBIP10/BBIP1). The Meckel–Gruber syndrome (MKS)/Joubert syndrome (JBTS) module is made of MKS1, Transmembrane Protein 231 (TMEM231), Transmembrane Protein 216 (TMEM216), Transmembrane Protein 67 (TMEM67), Transmembrane Protein 17 (TMEM17), Tectonic Family Member 1-3 (TCTN1-3), B9 Domain Containing 1-2 (B9D1-2), Coiled-Coil And C2 Domain Containing 2A (CC2D2A), Abelson Helper Integration Site 1 (AHI1), Intraflagellar (IFT) family of proteins and associated motor proteins (dynein Dynein Cytoplasmic 2 Heavy Chain 1 (DYNC2H1), Dynein Cytoplasmic 2 Light Intermediate Chain 1 (DYNC2LI1, WDR60, WDR34), Dynein Light Chain Tctex-Type 2B (TCTEX1D2), and kinesin) are critical components of ciliary transport and are often mutated in various ciliopathies. (Modified and redrawn using BioRender.com [60,61,62]).

Positional cloning and linkage studies were responsible for the identification of the first gene associated with NPHP in 1997 [45,46]. This was named *nephrocystin 1* (*NPHP1)*. Over the next decade, similar approaches allowed the identification of the next eight genes [47,48,49,50,51,52,53]. The advent of NGShas allowed more rapid identification of genes with causal roles in NPHP; the count currently stands at 26 [3,54,55]. Interestingly, these genes cover only about 30% of the disease spectrum, while almost two-thirds of NPHP genes remain unidentified [56,57]. Among these, mutations in *NPHP1* are the most common cause of NPHP, while the mutations in nephrocystins 2-20 (*NPHP2-20)* are said to account for less than 1% of total cases [54]. Almost all NPHP proteins are located in the transition zone or inversin compartment of the primary cilia, centrosome or are parts of intraflagellar complexes (IFT complex) (Figure 2), with the exception of *NPHP1L* and *NPHP2L* that localise to the mitochondria [55]. Despite residing in different chromosomes and belonging to a wide range of protein families with diverse cellular functions, NPHP proteins seem to work in modules that have designated roles in disease pathogenesis. Currently, four distinct NPHP modules have been identified, which include, *NPHP 14-8* module, *NPHP 2-3-9-16* module, *NPHP 5-6* module, and *MKS* module [58,59]. Investigations on mutations other than the predominant *NPHP1* gene could therefore provide novel insights into the pathogenic mechanism of this disease and potentially help in identifying novel NPHP genes. This is an important area of study given that currently the majority of NPHP causative genes are unidentified. 

NPHP genes can show phenotypic heterogeneity [63]. Mutations in a number of NPHP genes that present with syndromic clinical phenotypes showcase extrarenal manifestations such as Senior–Løken syndrome, Alström syndrome, Arima syndrome, Cogan syndrome, Joubert syndrome and related disorders, Meckel–Gruber syndrome, BBS, Ellis–van Creveld syndrome, Sensenbrenner syndrome, Jeune syndrome, and Mainzer–Saldino syndrome [55]. Although these syndromes are attributed to singular genes, there are cases where mutations within a particular NPHP gene have resulted in multiple phenotypes. These differences could be due to the site-specific mutations within the gene, although these mechanisms are not well understood. In some cases, digenic, oligogenic and triallelic inheritance have explained such phenotypic variances [64,65]. Triallelic inheritance has been proven in BBS, while oligogenic inheritance has been observed in clinical cases for genes, such as *NPHP 1, NPHP 5, NPHP 6, NPHP 8, NPHP 9, NPHP 11,* and *Tetratricopeptide Repeat Domain 21B (TTC21B)*, etc. [54,55].

## 5. NPHP Proteins

To date, mutations in 26 different genes have been found to result in NPHP-like phenotypes [3,54]. The proteins these genes encode (nephrocystins) belong to a diverse set of protein families with fundamental differences in structure and function. In the context of the disease however, each of these genes is associated with NPHP type 1 to 20 phenotype (in addition to six novel undesignated genes) to characterize the subtle phenotypic differences across the various NPHP genes.

### 5.1. NPHP1 

The *NPHP1* gene (2q12.3) encodes the protein nephrocystin-1, also termed nephrocystin [46,66]. It is the most clinically abundant mutated form of NPHP gene identified to date, and indeed, 85% of cases that carry the mutated form of this gene harbour a large homozygous deletion [67]. Spontaneous deletions and loss of function point mutations in *NPHP1* have also been identified [68,69]. NPHP1 interacts with p130 (Cas) and other components of cell-cell adhesion and cell-matrix signalling, such as focal adhesion kinase 2, filamin, and tensin [70]. In renal epithelial cells, NPHP1 with p130 localises to the focal adhesions and adherens junctions, facilitating cell basement membrane and cell-cell contacts, respectively [71,72]. It is targeted to the transition zone at the base of the primary cilia by casein kinase 2-mediated phosphorylation of phosphofurin acidic cluster sorting protein 1, which binds to nephrocystin and co-localises with it in the cilia [73]. In the cilia, it also interacts with nephrocystin-4 and protein fantom, linking it to inversin [73,74]. Mutations in NPHP1 has also been shown to affect phosphoinositide composition in the renal cilia 33306870. 

### 5.2. NPHP2 

NPHP type 2, resulting in the rare, yet clinically significant infantile NPHP (with or without *situs inversus*), is caused due to mutations in the *INVS* gene (9q21–22) encoding inversin [75]. The frequency of mutations in *INVS* in patients reaching ESRD under 2 years of age is as high as 78%. Although the distribution of cysts is corticomedullary, the overall kidney morphology resembles ARPKD with enlarged kidneys [54,76]. This is different from most other forms of NPHP which typically show normal or shrunken kidney sizes. 

Inversin plays an important role in cell adhesion through its interaction with nephrocystin-1 and nephrocystin-3 [53,77]. Inversin localises to various subcellular compartments in a cell-cycle dependent manner [78,79,80]. Particularly, inversin interacts with the β-tubulin that constitutes the ciliary axoneme and localises to the basal body of the primary cilium and centrosome during interface [44]. It associates with mitotic spindle fibres in mitosis and is found at the mid-body during cytokinesis [78]. In 2005, inversin was shown to have roles in cell-cycle regulation and the planar cell polarity required to maintain normal tubular development and morphology [81]. In the cilia, it acts as a molecular switch between non-canonical and canonical Wnt pathways. Defects in inversin renders the canonical Wnt pathway in a perpetually active state, leading to the improper apical basolateral polarity of the tubular epithelium.

### 5.3. NPHP3 

Mutations in the *NPHP3* (3q22.1) gene cause adolescent NPHP, associated with *situs inversus* and structural heart defects [77]. Mutations in this gene were identified in a large Venezuelan kindred in 2000 [82]. Mutations in the murine ortholog of *NPHP3* presented with renal cysts and were called the *pcy* model [53]. This murine model was responsive to treatment with a vasopressin receptor agonist that eventually led to the development of Tolvaptan as the first FDA approved treatment for ADPKD in the United States [83,84]. Complete loss of this gene has been associated with embryonic lethality, *situs inversus* and congenital heart defects in mice [77]. Truncating mutations in this gene in humans present with Meckel–Gruber syndrome (*situs inversus*, CNS malformation, congenital defects of heart, kidneys, urinary tract, polydactyly, and preauricular fistulas) [77]. NPHP3 interacts with inversin; INVS (NPHP2), serine/threonine-protein kinase Nek8; Nek8 (NPHP9) and Ankyrin repeat and SAM domain-containing protein 6; and ANKS6 (NPHP16) in the inversin compartment of the cilia [59]. 

### 5.4. NPHP4 

Homozygosity mapping and genome-wide linkage analysis led to the identification of mutations in *NPHP4* (1p36.31) in NPHP patients lacking mutations in *NPHP1, 2* and *3* genes [85]. Individuals with *NPHP4* mutation frequently present with a retinal phenotype [52,85,86]. The protein encoded nephrocystin-4 localises to primary cilia, basal body, and the cortical actin cytoskeleton in polarized cells [86]. Similarly to inversin, it localises to the centrosomes in dividing cells. Nephrocystin-4 has been shown to interact with cell-cell adhesion entities like p130cas, filamin, and tensin [86].

### 5.5. NPHP5 

Mutations causing truncations in the IQ-calmodulin-binding motif-containing protein-1 IQ (isoleucine (I) and glutamine (Q)) (*IQCB1)* gene (3q13.33) underlie NPHP cases with early-onset retinal degeneration (Senior–Løken syndrome) [51]. It encodes nephrocystin-5 or *IQCB1*, which, as the name suggests, contains an IQ domain that interacts with calmodulin [51]. It also forms a complex with retinitis pigmentosa GTPase regulator (RPGR), and defects in this gene result in X-linked retinitis pigmentosa. Interestingly, RPGR and nephrocystin-5 are both located in the connecting cilia (structural equivalents of primary cilia in photoreceptor cells), where they associate with calmodulin. They also localise to the primary cilia of renal epithelium [51]. 

### 5.6. NPHP6 

Truncating mutations in the centrosomal protein of 290 kDa (*CEP290) gene* (12q21.32) were identified as a novel gene in 2006, and as the most common cause of NPHP associated with Joubert Syndrome and related diseases [31]. It is also the leading genetic cause of retinal degeneration (Leber’s congenital amaurosis) [87]. Furthermore, it is implicated in a few cases of BBS [88]. It interacts with two other NPHP proteins, meckelin and coiled-coil, and C2 domain-containing protein 2A, and may cause other ciliopathies, including Meckel–Gruber syndrome [48]. This gene encodes the protein CEP290 (nephrocystin-6, also referred to as MKS4, KIAA0373, 3H11AG, JBTS5, SLSN6, LCA10, and BBS14), which is expressed in a cell-cycle dependent manner and localises to the mitotic spindle fibres and centrosomes [31]. CEP290 could potentially affect the cAMP pathway through its interaction with cyclic AMP-dependent transcription factor ATF-4 (ATF4/CREB2), which has been implicated as a vital transcription involved in cystogenesis [31,83]. Several studies report CEP290′s role in cell signalling, ciliogenesis and DNA damage responses [89]. Absence of this protein causes cell polarity defects in zebra fish models [25,31]. In human type 6 NPHP, mutations in *CEP290* present as renal cysts, retinal degeneration, and cerebellar defects [48]. 

### 5.7. NPHP7 

Mutations in the Zinc finger protein GLIS2 (*GLIS2)* gene result in NPHP type 7 [49]. It encodes the transcription factor Gli-similar protein 2, loss of which results in the overexpression of genes involved in epithelial to mesenchymal transition and has been associated with pronounced fibrosis, apoptosis, and cell senescence [49]. GLIS2 expression alongside pharmacological stabilizers of p53 has been shown to partially rescue the phenotype in a kinesin-like protein KIF3A (*kif3a*) null mice with renal cysts formed due to hyperproliferation, DNA damage and destabilised p53 [90]. The Wnt and hedgehog signalling pathway, which determines tissue patterning during embryogenesis, involves the GLI transcription factor [91]. GLIS2, being related to GLI, may employ similar pathways for cystogenesis. 

### 5.8. NPHP8 

*RPGRIP1L* gene mutations result in NPHP8 and are usually associated with Joubert or Meckel–Gruber syndrome [48]. It encodes the protein fantom that localises to the basal body and centrosome, interacting with NPHP4 and NPHP6 [92,93]. The *RPGRIP1L* gene shows multi-allelism, where two truncating mutations result in Meckel–Gruber syndrome while missense mutations lead to Joubert syndrome [92,94]. 

### 5.9. NPHP9 

NPHP type 9 is associated with all forms of NPHP (including infantile NPHP) [47]. It is caused by mutations in the *Nek8* (*NPHP9*) gene. Nek8 was first identified as an aetiological cause of NPHP in a murine model (*jck* mice) and in zebrafish [95]. In humans, Nek8 was first identified in a study on primary breast cancer, where it was found to be overexpressed [96]. In due course, human infantile NPHP patients with mutant *Nek8* were also identified, warranting its classification in the NPHP group of proteins [47]. Mutations in *Nek8* have also been described in a rodent model (the Lewis polycystic kidney (LPK) rat) of autosomal recessive cystic kidney disease [97,98]. 

Nek8 is a serine-threonine kinase with a broad cellular localisation profile. Nek8 resides in the proximal part of the ciliary shaft within the inversin compartment and interacts with other NPHP proteins [99]. It was also known early on that Nek8 localises to the nucleus [100]. Being from the Nek family of proteins, Nek8 is assumed to have a mitotic role, despite the fact that no clear evidence has been reported in support of this. Proteomics of renal cysts in *jck* mice revealed upregulation of vimentin, sorcin, and galectin-1 [101], hinting at a possible role of Nek8 as a check point regulator of mitotic spindle, through interactions with the polycystin complex. More recently, Nek8′s nuclear localisation has been more extensively corroborated with its functional role in the maintenance of genome stability through its physical and functional interaction with components of ATR-mediated DNA damage repair [102,103]. Thus, loss of function of Nek8 has linked replication stress to functions of the primary cilia and genome stability as a path to developing ciliopathies, such as cystic kidney disease. 

### 5.10. NPHP10-20 and Other Putative NPHP Genes

Positional cloning technologies were pivotal in identifying causal NPHP mutations in NPHP1-9 genes over the span of 15 years (1993–2008) [25]. With the advent of NGS, identification of new NPHP genes (*NPHP10-20*) alongside putative NPHP genes such as X-Prolyl Aminopeptidase 3 (*XPNPEP3/NPHP1L)*, Solute Carrier Family 41 Member 1 (*NPHP2L/SLC41A1)*, TRAF3 inter-acting protein 1 (*TRAF3IP1)*, Abelson Helper Integration Site 1 (*AH11/JBTS3)*, Coiled coil and C2 domain containing 2A (*CC2D2A/MKS6/JBTS9)* (novel or originally associated with syndromic ciliopathies) were rapidly linked to NPHP phenotypes [35,56,58,59,104,105,106,107,108,109,110,111,112,113,114,115,116,117,118,119,120,121,122,123]. However, the molecular pathogenesis of these mutations and the role of these proteins in context of NPHP are poorly characterized. The NPHP phenotypes associated with all known NPHP genes and proteins are detailed in Table 1.

## 6. Biochemical Pathways

The years 1995 and 1996 marked the discovery of *Polycystin 1 (PKD1)* and *Polycystin 1 (PKD2)* as the first genes to be associated with cystic kidney disease through positional cloning [126,127]. The proteins encoded by these genes, i.e., polycystin 1 (PC1) and polycystin 2 (PC2), were shown to have roles in cell-cell interaction and cell cycle with no clear mechanistic link to cystogenesis. Mutations in *Tg737* encoding polaris was then identified as the causative mutations in the Oak Ridge polycystic kidney mouse (*orpk)* model of ARPKD [128,129,130,131]. This mutation resulted in shunted primary cilia in renal epithelial cells, and animals with this disease presented with multi-organ defects in the liver, pancreas, and brain, and had skeletal abnormalities, besides cystic lesions in the kidneys [132,133,134,135,136,137]. It was only in 2002 that the link to cilia was evident when PC1, PC2, and polaris were shown to co-localise in the primary cilia of the renal epithelium [138]. Eventually, other mutations leading to cystic kidney diseases were also mapped to ciliary proteins. In 2003, the landmark paper by Otto et al. identifying *INVS* mutations in NPHP [44] led to the proposition that all causal genes of cystic kidney diseases in humans, rodents or zebrafish must be expressed in the primary cilium, basal body or centrosome [139]. It is evident from the previous sections that this axiom holds ground even two decades after it was originally proposed, noting the rare exceptions for XPNPEP3, which was identified as a mitochondrial protein [120], and SLC41A, for which a ciliary location has not yet been confirmed [54]. 

The primary cilium recruits several specialised receptors and ion channels that have been associated with biomechanical and biochemical circuits specific to the cilia [62]. Given that renal cysts are a common feature resulting from dysregulation or mislocalisation of ciliary proteins [139,140], much of what is known about ciliary cell signalling has been through studies on animal models of PKD. Clearly, therefore, many of these studies set the foundation for existing and emerging therapeutic strategies for treating cystic kidney diseases. Although the complete spectrum of bioprocesses modulated by the renal cilium is not well understood, signalling pathways that are considered cilia centric are discussed below. Their role and potential in the development of therapeutic interventions, where relevant, have also been reviewed in later sections. While the existing literature on biomolecular processes governing cystic kidney diseases are PC1 and PC2 centric, they may overlap with the pathogenic mechanisms of other cystic diseases, such as ARPKD, NPHP or MCKD and will be discussed in depth in this section. 

### 6.1. Mechanosensation in Renal Cilia

**Figure 3:**
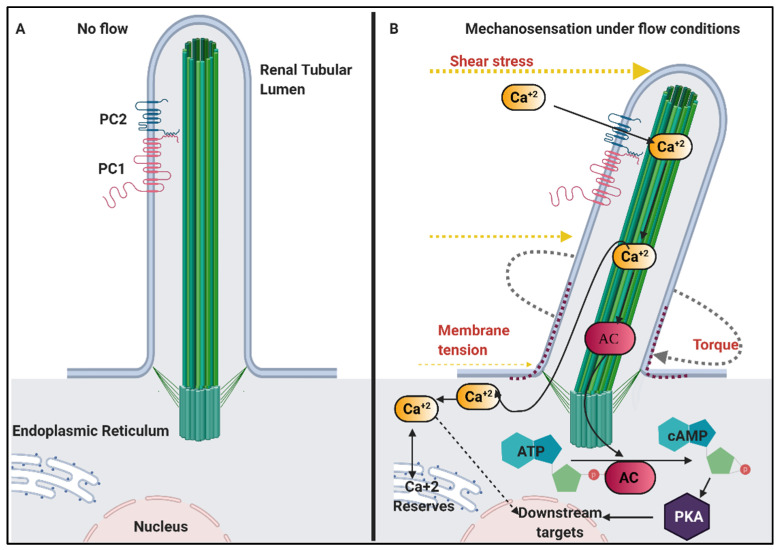
Molecular processes governing mechanosensation in primary renal cilia: (**A**) The renal cilium projects into the lumen of the renal tubule where it can sense physical forces resulting from luminal flow. The polycystin complex (made of polycystins 1 and 2 (PC1/PC2)) located in the ciliary membrane helps transducing these mechanical cues. (**B**) The cilia senses mechanical forces like shear stress, membrane tension or torque under flow conditions. This stimulus is hypothesised to activate PC2, resulting in an influx of Ca^2+^ which in turn activates adenyl cyclase (AC). AC converts adenosine triphosphate (ATP) to cyclic AMP (cAMP). cAMP acts as a signalling molecule for protein kinase-A (PKA) which modulates the expression of downstream proteins. The influx of Ca^2+^ also regulates intracellular calcium homeostasis, thereby affecting the regulation of calcium reserves from the endoplasmic reticulum. (Modified and redrawn using BioRender.com [150,151]).

Zimmermann was the first to propose the concept of primary cilia in renal tubules acting as flow-sensors [141]. The first piece of evidence supporting this hypothesis came over 100 years later from Kenneth Spring’s laboratory in Denmark, where a transient increase in levels of intracellular Ca^2+^ was demonstrated in cultured renal epithelial cells, resulting from fluid flow-induced deflection of the primary cilia [142,143,144]. Upon deciliation, this Ca^2+^ response was revoked, emphasising the importance of the primary cilia in this mechanical, flow-induced elevation of cytosolic Ca^2+^ (Figure 3). Studies by Liu et al. [145] further demonstrated that calcium signals were severely abrogated in the absence of luminal flow in microperfused collecting ducts of 2-week old *orpk* mice with stunted cilia. PC1 and PC2 both localise to cilia and their dysfunction in renal cells have similar repercussions on flow-mediated intracellular Ca^2+^ levels, even in the presence of a fully formed cilium [146]. This led to the hypothesis that PC1 senses the mechanical stimuli (flow) and activates PC2 channels, mediating an influx of Ca^2+^ ions in the cell. Deficiencies in proteins such as Nek8, fibrocystin/polyductin (FPC), and the transient receptor potential cation channel subfamily V member 4 (TRPV4), which is known to interact with PC2, also result in compromised mechanotransduction, thus further supporting this hypothesis [147,148,149]. In consideration of the fact that mutations in PC1 and PC2 cause renal cysts, loss of cilia-mediated mechanotransduction, leading to aberration in Ca^2+^ signalling, was proposed to be the driving force behind renal cystogenesis. This theory, however, sparked a debate in the field of molecular nephrology.

Conditional mutations in *PKD1* and *IFT88* mice revealed that cilia dysfunction induced before postnatal day 12 (P12) resulted in a rapid onset of cystic kidney disease whereas, cystogenesis was remarkably prolonged if a ciliary loss occurred after P12 [152,153,154]. This was difficult to explain if cystogenesis were merely meant to be a function of the loss of mechanotransduction. Furthermore, the heterogeneity across various cystic phenotypes resulting from cystoproteins interacting with PC1/PC2 also posed a question on this hypothesis. For example, the presence of glomerular cysts in Nek8 null mice that die at birth [147] compared to the *Nek8^jck/jck^* phenotype, as seen in adult mice, that resembles ARPKD, was hard to explain through mechanical shear stress triggers alone [155,156,157]. Moreover, Trpv-4 deficient mice and zebrafish with compromised mechanotransduction lacked the presence of renal cysts [149]. This is also the case in certain human NPHP phenotypes [158]. However, in *Polycystic Kidney And Hepatic Disease 1/polyductin* ((*PKHD1),* mutated in ARPKD) mutant *pck* rats, activation of an otherwise deficient Trpv-4 protein helped attenuate cystogenesis [159]. Thus, mechanotransduction seemed to function more as a regulator than an initiator of cystogenesis.

Techniques developed to visualize Ca^2+^ influx into the cell over the last decade have contributed to some extent in resolving this enigma [160,161,162]. Jin et al. used genetically encoded Ca^2+^ indicators fused to ciliary localised proteins and showed that Ca^2+^ levels in the cilium increase upon its mechanical bending, which is followed by a subsequent increase in cytosolic levels of Ca^2+^ [162]. Other studies corroborate the mechanotransduction of fluid shear stress in renal cilia converted into Ca^2+^ signals along with the role of PC1 and PC2 proteins in this process [163]. However, patch clamping studies by DeCaen et al. in 2013 illustrated that these ion currents were generated by Polycystin 1 Like 1, Transient Receptor Potential Channel Interacting (PKD1L1) and Polycystin 2 Like 1, Transient Receptor Potential Cation Channel (PKD2L1) [164]. In these experiments, PC1 and PC2 activities were undetectable. Other experiments disrupting the ciliary tip also allowed transient and insignificant increases in Ca^2+^ levels in the cytosol [165]. This opened possibilities for other protein candidates with mechanosensing properties, other than PCs, devoid of Ca^+2^ signalling properties to be investigated [166,167]. A caveat in these contradicting studies however was, besides flow, other modes of PC1 and 2 channel activation and sensing were not excluded [168]. Eventually, a study by Kim et al. in 2016 proposed one such mechanism of PC2 activation via Wnt ligands [169]. The layers of unknown biochemical regulations governing roles of primary cilium as a mechanotransducer require further studies, the intricacies of which have been reviewed extensively by Ferreira and colleagues [151]. Therefore, despite the debate, this pathway continues to remain a critical variable in the study of cystic pathologies.

### 6.2. Vasopressin, cAMP and Ca^2+^ Signalling in Renal Cilia

**Figure 4:**
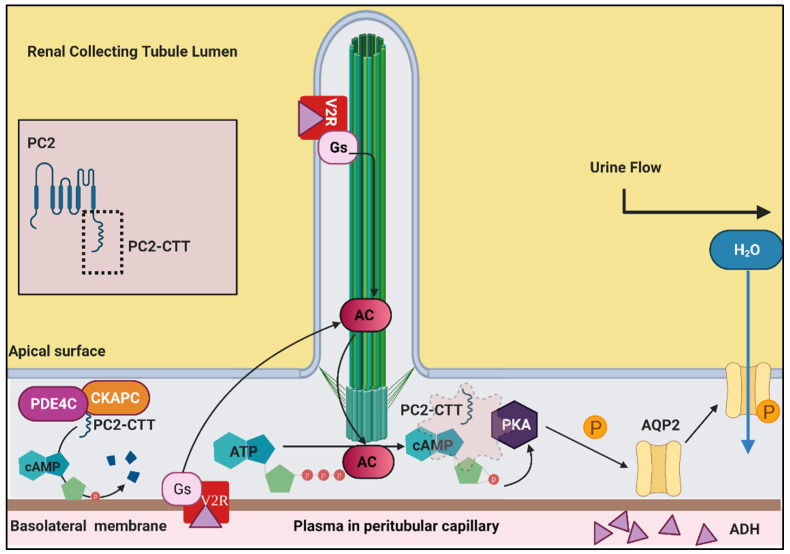
Vasopressin, cAMP and Ca^2+^ signalling in renal cilium: Vasopressin/antidiuretic hormone (ADH) is released by the hypothalamus upon sensing hyperosmolar states to facilitate reabsorption of water to maintain homeostasis. Circulating ADH binds to the vasopressin type-2 receptor (V2R) in the kidney which activates adenyl cyclase (AC). AC converts ATP to cAMP which in turn triggers protein kinase A (PKA). The PC2 C-terminal tail (CTT) plays a pivotal role in providing a scaffold for these protein interactions. PKA phosphorylates aquaporin-2 (AQP2) which is then trafficked to the apical surface (towards the tubular lumen), which allows water absorption from urine. The PC2-CTT also interacts with phosphodiesterase 4C (PDE4C) and other components of a ciliary A-kinase anchoring protein complex (CKAPC) [composed of adenylyl cyclase 5/6 (AC5/6), A-kinase anchoring protein 150 (AKAP150), and PKA] and facilitates cAMP catabolism. (Modified and redrawn using BioRender.com [150,179]).

Antidiuretic hormone (ADH, also called vasopressin) is a nonapeptide synthesised in the hypothalamus of the brain and is mainly responsible for maintaining tonicity and water homeostasis in the body [170]. Osmoreceptors in hypothalamic neurons detect changes in blood osmolarity (as tiny as 2 mOsm/L) that trigger vasopressin release in hyperosmolar states [171,172]. Released vasopressin targets the vasopressin type-2 receptor (V2R) in the kidney (Figure 4) [173]. V2R localises in the primary cilia of the renal epithelium and basolateral membrane of the collecting ducts that guides the kidneys to reabsorb water and concentrate the urine being produced. The V2R functions as a GPCR, which, upon activation, activates adenyl cyclase (AC) [173,174]. Adenyl cyclase converts ATP to cAMP, with cAMP acting as a potent signalling molecule that eventually activates protein kinase-A (PKA) (Figure 4). PKA phosphorylates the water channel aquaporin-2 (AQP2) [175,176,177]. Upon phosphorylation, AQP2 is trafficked to the apical surface of the cell, which is the luminal side of the tubular cells (Figure 4) [178]. This allows the uptake of water back into the blood stream, concentrating the urine and returning blood osmolarity to normal. 

In the context of cystic kidney diseases, PC2 has been found to play a central role in regulating not only cAMP but Ca^2+^ levels in the cell. It is the PC2 C-terminal tail that forms a scaffold with A-kinase, and the anchoring protein allows the interaction of PKA and AC that is essential for cAMP synthesis (Figure 4) [180]. PC2 also helps recruit the phosphodiesterase 4C essential for cAMP catabolism, thus playing an overarching role in cAMP regulation [180]. *PKD2* homozygous knockouts exhibit increased levels of cAMP [181], however, overexpression of the wildtype PC2 result in decreased levels of cAMP, implicating its Ca^2+^ channelling properties in cAMP regulation [60,182]. The exact mechanisms linking cAMP and Ca^2+^ require further investigation. Gene dosage seems to play an important role in determining cystic phenotypes, which complicates deducing these mechanisms. For example, CaV1.2, a voltage-gated L-type calcium channel that localises to the primary cilia, is absent in *PKD1* and *PKD2* homozygous knockouts. Interestingly, *PKD1* heterozygote mice do not present with renal cysts, however, the deletion of this Ca^2+^ channel in *PKD1* heterozygotes can result in the formation of extremely large renal cysts in a few of the cases [183]. 

Despite this variability, targeting the cAMP pathway has resulted in the first food and drug administration (FDA) approved therapeutic intervention for clinically managing early-stage ADPKD patients to date [84]. Tolvaptan is a V2R antagonist that reduces cAMP levels in cells, thereby reducing the rate of cyst growth in kidneys [184]. This drug has also been approved for ADPKD management in Australia, Japan, Canada, Europe, and the USA. However, there are certain concerns around overall tolerance, safety, and prolonged use of this drug [185,186]. Tolvaptan administered along with activators of somatostatin receptors has shown to be more effective in *PKD1^RC/RC^* mouse than either drug alone [187]. Knockout of other cAMP catabolizing enzymes in *PKD2* mice models showcase aggravated cystogenesis, emphasising a direct molecular mediation of cystic phenotype through this pathway [188]. This evidence could allow researchers to harness the translational potential of proteins belonging to this pathway to reduce the cystogenic burden of diseases such as PKD and NPHP.

### 6.3. PI3K/Akt/mTOR Signalling in Renal Cilia

The phosphoinositide 3-kinase (PI3K), AKT serine/threonine kinase (AKT) and mammalian target of rapamycin (mTOR) are kinases that incorporate a wide range of upstream signals to regulate fundamental cellular processes, such as translation and protein synthesis, cell metabolism, cytoskeleton rearrangement, redox sensing, cell growth, proliferation and senescence [189]. Although these are kinases that mediate specific signalling cascades, there is a huge degree of overlap that is relevant in the context of cystic kidney diseases, especially when considering the mTOR branch [190,191,192].

This range of processes in the mTOR pathway is primarily mediated through two complexes: mTORC1 and mTORC2 [193]. In regular physiological conditions, nutrient availability stimulates mTORC1, which facilitates phosphorylation of its down-stream effector p706S (S6K) (activated) and 4E-BP1 (inhibited) that regulates protein synthesis and cell growth [194]. AMP-activated protein kinase (AMPK) acts as an “energy sensor” within cells to inhibit mTORC1 [194,195]. Boehlke et al. showed that these processes were cilia dependent, ablation of which resulted in the upregulation of the mTOR pathway and defects in cell size [195]. It is therefore not surprising that the mTOR pathway is activated in several PKD animal models and cyst linings of human ADPKD patients [192,196,197]. 

**Figure 5:**
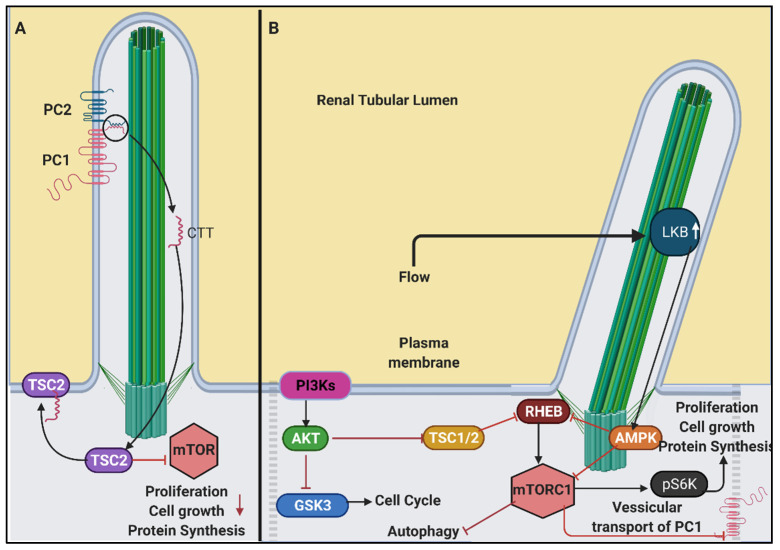
Regulation of phosphoinositide 3-kinases (PI3K)/protein kinase B (Akt)/mammalian target of rapamycin (mTOR) signalling in renal cilia: (**A**) The PC1 CTT oversees the membrane trafficking of tuberculosis sclerosis complex 2 (TSC2), essential for homeostatic (negative) regulation of mTOR pathway. (**B**) Graphical representation of PI3K-AKT kinases and its link to mTOR pathway. Upon flow, the expression of tumor suppressor kinase Liver Kinase B1 (LKB1) increases which activates AMP-activated protein kinase (AMPK). AMPK negatively regulates mTORC1. Expression of downstream effectors like ribosomal protein S6 kinase B1 (pS6K) is enhanced upon mTOR activation. This leads to proliferation, cell growth, and increased protein synthesis. (Modified and redrawn using BioRender.com [150]).

It has been shown that the C-terminal tail of PC1 undergoes cleavage due to a mechanical stimulus [198,199]. This regulates the membrane localisation of tuberculosis sclerosis complex 2 (TSC2), which is an inhibitor of mTOR (Figure 5) [198]. Dysfunction or absence of PC1 affects the processing of its C-terminal tail, which in turn affects the localisation of TSC2, resulting in an activated mTOR [190,198]. In the presence of fluid flow, renal cells with ablated cilia have a larger cell size [195]. This effect was attributed to defects in the mTOR pathway. Interestingly, this was not due to irregular PC1 cleavage or PC2 mediated by calcium or signal, but rather due to reduced activity of tumour suppressor kinase Liver Kinase B1 (LKB1) in response to flow. LKB1 phosphorylates AMPK and localises to the ciliary base, which suppresses mTORC1 (Figure 5) [195]. In LKB1 deficient cells, pAMPK levels are reduced in cilia, and remain unaltered in the cytoplasm, in the presence of flow. These conditions cause increased cell size due to activated mTOR. The cells with *PKD2* knocked down expression do not show elevation of the mTOR downstream effectors, such as pS6K, nor does it show any change in cell size in the presence of flow [195,200]. This suggests that flow-mediated suppression of mTOR in cells is independent of Ca^+2^ signalling pathways [195]. Besides mechanotransduction triggered mediators, ubiquitination of c-Met (hepatocyte growth factor receptor (HGFR)) has also been identified as a regulator of this pathway [201]. Lack of ubiquitination of HGFR activates the mTOR pathway in PKD mouse models [201], while cystic phenotype is suppressed on the administration of c-Met inhibitors [202]. 

PI3K/Akt signalling is prominent in renal cancers and cysts arising due to homozygous inactivation of the Von Hippel Lindau (VHL) [203] and Birt–Hogg–Dubé (BHD/FLCN) [204] tumour suppressor genes. However, the role of the Phosphoinositide 3-Kinase/Phosphatase and tensin homolog (PI3K/PTEN) branch may be more auxiliary than causal in the broad context of cystogenesis. This is because, while PTEN inactivation alone is insufficient in causing renal cysts, in the presence of a *VHL*-null genetic background, it increases renal cysts and tumours in mice [203]. Besides Tuberous sclerosis complexes ((TSCs): TSC1 and TSC2), GSK3β is a shared downstream effector of PI3K and mTOR pathways that are directly involved in ciliogenesis in a non-canonical manner [205,206]. Concomitant loss of GSK3β and VHL results in renal cysts [207]. Inositol phosphatase-5-phosphatase E (INPP5E) is another downstream effector of the PI3K pathway, which, when deficient, results in structural defects in cilia and cyst formation in the kidney [208]. Recent studies have shed light on several other non-mitotic intermediates of these pathways with implications in intraflagellar trafficking (tubby-like protein 3: TULP3) [209], ciliary vesicular trafficking and apical-basal polarization (PIK3C2A) [208,210] that could contribute to cystogenesis. PI3K/Akt pathway intermediates are therefore being explored as targets for therapeutics.

Multiple pre-clinical studies have highlighted the translational potential of mTOR inhibitors on PKD animal models [192,211,212,213,214,215]. These strategies have, however, not seen comparable success in human ADPKD trials [216,217,218]. Most commonly, rapamycin and rapalogs (analogs/derivatives of rapamycin) used in pre-clinical studies did not show a significant difference in total kidney volume [217] or overall kidney function compared with those receiving standard care or a placebo [218]. In some cases, long term use (1 year) of rapamycin showed a greater decline in renal health than those receiving standard care and was deemed unsafe [216]. The kidney expresses high levels of folate receptors; hence, reducing effective rapamycin dosage (and toxicity) by conjugating it with folate may result in better targeting of kidney cells (instead of the spleen) via folate-mediated endocytosis, as has been shown in PKD mice models [219]. Furthermore, new classes of mTOR inhibitors, including 2-(4-amino-1-isopropyl-1H-pyrazolo[3,4-d]pyrimidin-3-yl)-1H-indol-5-ol dihydrate or PP242, that reversibly inhibits both complexes of mTOR, have been tested to reduce the feedback from mTORC2 resulting from rapamycin-mediated inhibition of mTORC1 [220]. Alternatively, upstream targets of mTORCs, such as AMPK regulation, is seen as a more effective pathway for reducing cystogenic burden. Metformin, a drug known to activate AMPK, suppressed the mTOR pathway in *PKD1* mouse mutants, attenuating cystogenesis [200,202]. However, there were concerns that the metformin dose required to therapeutically activate AMPK in the human kidney might be much larger than was clinically achieved [221,222]. A recent study reported the use of combination therapies to test the synergistic effects of metformin alongside salsalate [223]. It was found that salsalate has a similar attenuating effect on cystogenic burden in PKD mutant mice when used in combination with metformin [223]. These combination strategies or identification of novel drugs targeting the mTOR pathway may help develop more effective therapies for PKD.

### 6.4. Jak/Stat and Inflammatory Signalling in Renal Cilia

Janus kinase (Jak)-signal transducer and activator of transcription (Stat) represent one of the most evolutionarily conserved and fundamental pathways governing development and homeostasis in mammals [224,225]. This pathway is triggered by signals from cytokines and growth factors [225]. Once activated, the mediators of this pathway regulate the transcription of processes involving cell proliferation, migration, development, differentiation, and most notably, immune responses [225]. While Jak-Stats are critical for kidney development, their role in renal disease is prominent in the immune response generated from kidney injury, loss of flow conditions, obstruction, cysts, or a combination thereof [226,227,228,229]. This causes an increase in macrophage infiltration and cytokine levels within the kidney [230,231].

**Figure 6:**
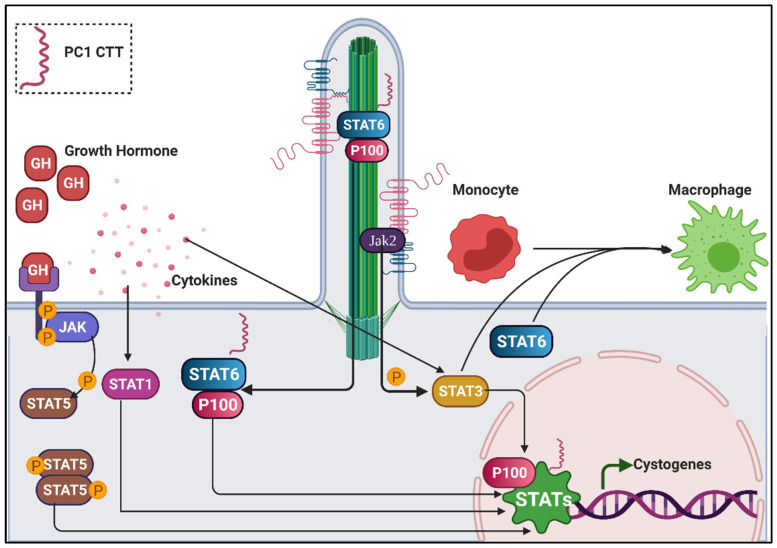
Janus kinase (Jak)/signal transducer and activator of transcription (Stat) pathway in cystogenesis: In the absence of flow, ciliary PC1 activates Jak2 which STAT3. Lack of flow also triggers PC1 CTT cleavage which activates STAT6 and NF-kappaB p100 (P100). STATs along with P100 translocates to the nucleus mediating transcription of genes required for cell proliferation, migration, development, differentiation, and immune responses. Cytokines released due to renal injury and immune response triggers may activate STAT3 and STAT1. Growth hormone (GH)-mediated signal transduction activates STAT5. STAT3 and STAT6 also facilitate monocyte differentiation to macrophage. These translocate to the nucleus mediating the expression of Jak/Stat downstream targets. (Modified and redrawn using BioRender.com [150,189]).

While this signalling cascade covers a broad range of upstream effectors, PC1 is its connecting link to the primary cilia. PC1 achieves this through multiple mechanisms. In one mode, PC1 in the cilium directly interacts with Jak2 kinase in the absence of flow (Figure 6) [232]. Jak2, along with the membrane-bound C-terminal tail of PC1, phosphorylates STAT3. Activated STAT3 translocates to the nucleus, triggering cystogenes (Figure 6). Loss of fluid flow in the nephron releases the C-terminal tail from its membrane-bound state. The PC1 C-terminal tail then activates P100 and STAT6 in the basal body of the cilium, activating cell proliferation via the interleukin pathways (Figure 6) [233]. The C-terminal tail of PC1 also translocates to the nucleus (Figure 6). Here, it enhances the activity of the cytokine activated STAT1 to facilitate p21-mediated antiproliferation and growth arrest [234]. Overexpression of PC1 has shown to cause increased phosphorylation in STAT1 [234]. Mutant mouse embryos lacking *PKD1*, conversely, have decreased levels of activated STAT1, validating its protective function against cystic growth [234]. Recently, Fragiadaki et al. reported a novel role of STAT5 in proliferation, as well as the regulation of growth hormone (GH) signalling, in the context of PKD (Figure 6) [235]. This explains why the administration of Tolvaptan along with somatostatin (GH-inhibiting hormone) receptor activators have shown improved efficacies in pre-clinical settings [187]. However, as is the case with other signalling pathways, inhibiting an upstream effector specific to the PKD axis may substantially help to reduce the toxicity emerging from the off-target effects of these broad, fundamental, and omnipresent pathways (e.g., metformin vs. rapamycin in targeting mTOR pathway). In light of recent findings, STAT5 SH2-domain inhibitors have been considered as promising candidates to target the GH–GHR–Jak–STAT5 axis in comparison to global GH or Jak inhibitors [236].

Activation of the Jak/Stat pathway in nephrons also triggers a series of immune responses. Chemoattractants, such as monocyte chemoattractant protein-1 of the C-C chemokine family and C-X-C motif chemokine 16, are secreted by the epithelial cells lining the renal cysts, which recruit monocytes and neutrophils to combat the renal damage [237,238]. In the kidney, STAT3 and STAT6 promote monocytes differentiation and polarizes them to form M1(pro-inflammatory) and M2 (pro-fibrotic) lineages of macrophages (Figure 6) [230]. A study by Karihaloo et al. showed a reduction in cystic phenotype upon macrophage depletion in PKD mouse models [230]. Evaluation of the therapeutic value of these pathways, however, requires detailed investigations on immune responses generated in cystic kidney diseases, such as PKD and NPHP.

### 6.5. Wnt Signalling and Planar Cell Polarity Defects in Renal Cilia

Wnt signalling is a highly conserved pathway dictating the fate of stem cells, development of conjoining cells throughout embryonic and foetal development, and organ patterning in vertebrates [239]. It is also responsible for regulating coordinated proliferation, cell polarization, branching morphogenesis and basement membrane synthesis, playing a critical role in renal development [239,240].

**Figure 7:**
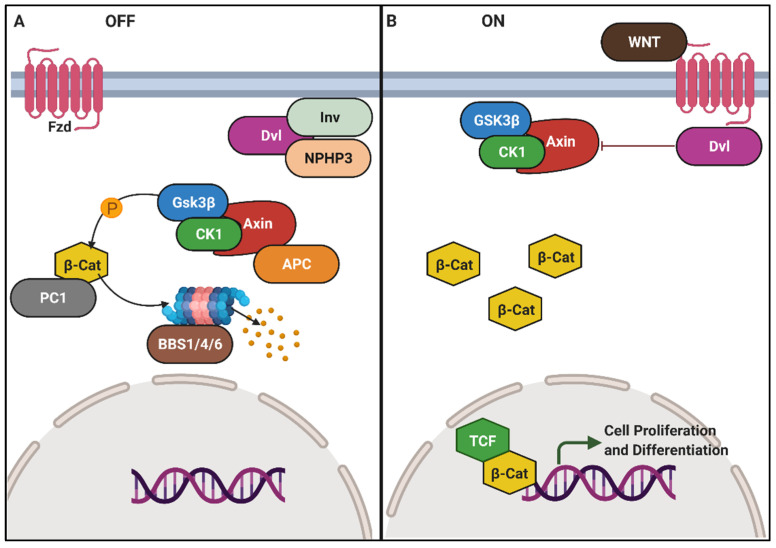
Canonical wingless/integrated (Wnt) signalling pathway: (**A**) In the absence of Wnt ligands, dishevelled (Dvl) forms a destruction complex with Axin, Glycogen synthase kinase-3β (Gsk3β), casein kinase 1 (CK1), adenomatous polyposis coli (APC). This destruction complex phosphorylates β-catenin (β-Cat) and triggers its proteosomal degradation. (**B**) In presence of Wnt ligands, it binds to frizzled (Fz) receptors. This phosphorylates Dvl thereby disassembling the destruction complex. β-Catenin (β-Cat) evades proteosomal degradation and is transported to the nucleus where it dimerizes with T-cell factor/lymphoid enhancer factor (TCF). This triggers expression of cell proliferative and differentiation genes. (Modified and redrawn using BioRender.com [150]).

The Wnt signalling is triggered when Wnt ligands (a large family of secreted glycoproteins) bind to the Frizzled (Fz) receptor (Figure 7). Thereafter, the presence of co-receptors dictates the path taken by this cascade into canonical (β-catenin-dependent) [241] or non-canonical (β-catenin dependent) directions. The canonical Wnt pathway involves the Fz and lipoprotein receptor-related protein-coreceptors. Upon binding of the Wnt ligand, the Fz–lipoprotein receptor-related protein ligation leads to the phosphorylation of dishevelled (Dvl) (Figure 7). Dvl disassembles the destruction complex (made of Axin2 acting as a scaffold, along with APC, CK1, and GSK3β) that phosphorylates β-catenin [241,242]. With the Dvl-mediated inhibition of the destruction complex, stabilized β-catenin evades proteasomal degradation and translocates to the nucleus and dimerizes with TCF (Figure 7) [243,244,245]. This acts as a transcription factor for genes, such as *Lef1, Axin* and *c-myc,* which are key regulators of cell proliferation and differentiation [242].

The non-canonical Wnt pathway, on the other hand, oversees tissue organization and morphogenesis through several signalling branches [81,246,247]. Planar cell polarity (PCP) is probably the most relevant branch in the context of cystic kidney diseases. PCP signalling comprising of oriented cell division and convergent extension is mediated through three classes of molecules, the upstream Fat and Dachsous (Ds) [248], PCP core proteins (Fz, Dsh/Dvl, Strabismus/Van Gogh (Stbm/Vangl), Prickle (Pk), Flamingo/Starry Night (Fmi/Stan), and Diego (Dgo)) [246] and downstream PCP effectors (Inturned (Int), Fuzzy (Fy), and RhoA, etc.) [240,249]. Through mechanisms unknown, upstream signals prompt the asymmetric distribution of PCP core proteins, causing PCP effector-mediated cytoskeletal reorganization [250,251]. Such modulations cause an asymmetric distribution of cellular proteins and the positioning of the basal body, that eventually determines cell polarity and orientation [252].

Several components of the Wnt pathway, including β-catenin, Dvl3 and adenomatous polyposis coli, localise to the cilia. Furthermore, mutants of ciliary proteins, such as BBsome, and components of IFT, including IFT88, AHI1 and Kif3a, show increased Wnt activity [253]. However, the functional roles of cilia in Wnt signalling are not clear. This is mainly due to pleiotropy and lack of canonical phenotypes across ciliary mutants. For example, IFT mutant embryos show enhanced hedgehog, not Wnt signalling [254]. Ciliary regulation of Wnt in some cases is also reported to be gene dose-dependent [255], which may, in turn, be organ dependent.

There are several other points of intersection between the Wnt pathway and kidney diseases, especially in the context of embryonic kidney development, organ morphogenesis, repair, cystogenesis, and ciliary biomechanical and biochemical mechanisms [240,256,257,258]. For instance, fluid flow regulates Wnt signalling in Inv (NPHP2) mutant IMCD cells [81]. Inversin was overexpressed in these cells and was found to antagonize canonical Wnt (through Dvl inactivation), promoting the PCP pathway, and thereby acting in the same manner as a switch [81]. In-vivo however, *inversin* null mouse kidneys were found to have normal canonical Wnt signalling [259]. Flow has been shown to regulate Wnt signalling in mouse embryonic nodes by Wnt3-mediated asymmetric decay of *Cerl2* mRNA [260]. This is crucial for the determination for the left–right axis.

**Figure 8:**
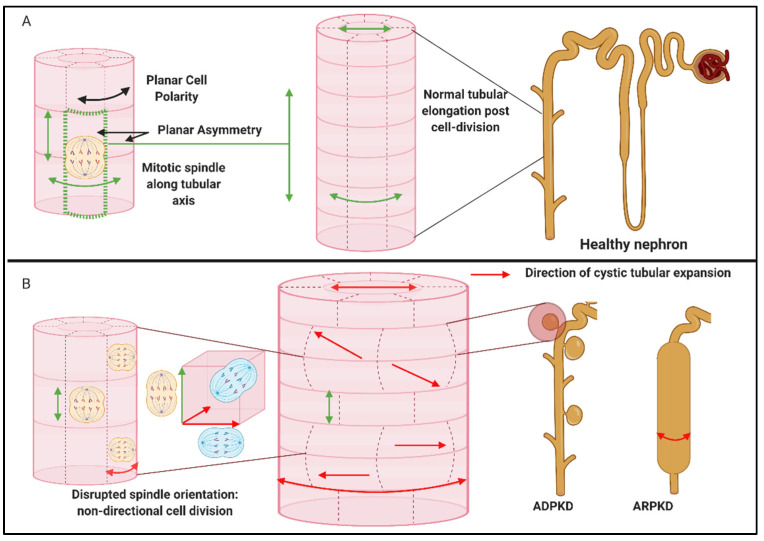
Planar cell polarity in cystogenesis: (**A**) Healthy renal tubular cells have a mitotic spindle aligned along the tubular axis resulting in planar asymmetry during cell division. Elongation thereby occurs uniformly across the tubular axis. (**B**) Abnormal planar cell polarity in renal tubular cells, as observed in polycystic kidney disease (PKD), involves disrupted spindle orientations which lead to non-directional cell division. Effectively, this leads to cystic expansion of renal tubules. (Modified and redrawn using BioRender.com [265,266]).

Wnt signalling also plays an important role in maintaining renal tubular structure through oriented cell division (Figure 8) [261]. This process ensures dividing cells align along the anterior–posterior axis of the nephron during early nephron development. Misoriented cell-division was proposed to cause cystogenesis, as it was observed in PCP mutants, such as *Fat4* and *Dchs1* [262,263]. In a study published by Kunimoto et al., the role of PCP signalling in renal tubules polarization during proliferative stages was fortified [264]. In their study using mice models, PCP mutations were found to act independently and in parallel to affect oriented cell division [264]. In isolation, PCP signalling only affected renal tubular diameter without inducing cysts [264]. This study established that PCP as a standalone is not cystogenic.

In a separate context, activation of noncanonical Wnt is accompanied by a transient increase in levels of intracellular Ca^2+^ following Fz activation [267]. This mechanism is poorly characterized. However, a study by Kim et al. added a novel paradigm to Ca^2+^ signalling, where Wnts were shown to bind to PC1 and induce PC2 dependent Ca^2+^ influx [169]. This study provided a hugely relevant and novel pathological arm of Wnt signalling to PKD.

### 6.6. Hedgehog Signalling in Renal Cilia

**Figure 9:**
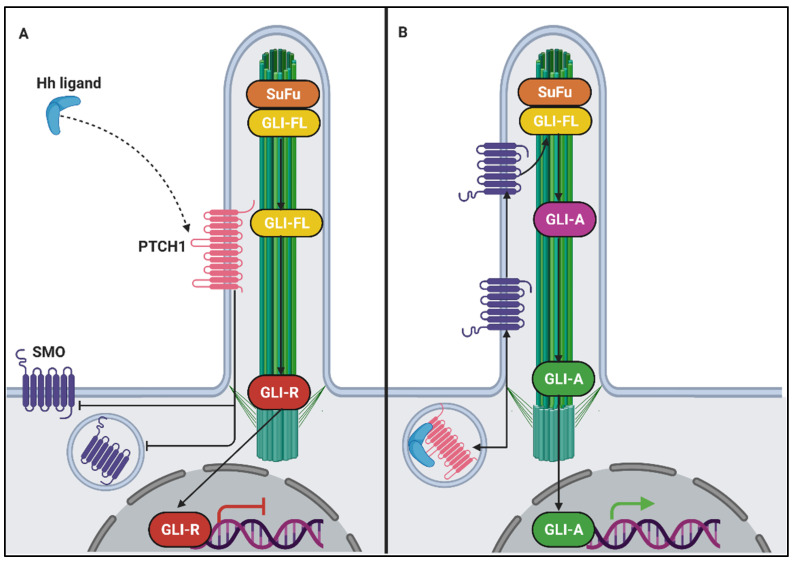
Hedgehog (Hh) signalling: (**A**) In an unbound state, patched (PTCH1) is localised in the cilia. In the absence of bound Hh ligands, full-length GLI family zinc finger 3 (GLI3) protein (GLI-FL) is proteolytically cleaved to form a repressed form of GLI3 (GLI3-R) which represses the expression of downstream genes controlled by Hh pathway. (**B**) When Hh ligands bind to PTCH1, it translocates out of the cilium. Simultaneously, smoothened (SMO), translocates and enriches into the ciliary compartment. SMO activates the GLI transcription factors GLI-A (made of GLI1 and GLI2). GLI-A translocates into the nucleus where its dynamic balance with GLI-R determines the output of downstream Hh genes (Modified and redrawn using BioRender.com [150]).

Hedgehog (Hh) signalling is one of the critical pathways to establishing body pattern, development, tissue differentiation, homeostasis, cell proliferation and growth in vertebrates. It is also one of the first and therefore most extensively characterized signalling pathways associated with primary cilia and its components [268]. In a nutshell, Hh ligands bind to a transmembrane receptor called Patched (PTCH1), normally located in the cilium. Hh bound PTCH1 initiates signalling and translocates out of the cilium (Figure 9) [269]. Simultaneously, smoothened (SMO), a transmembrane signal transducer, is enriched and subsequently activated within the ciliary compartment [270]. Once activated, SMO results in activation of GLI transcription factors (GLI1/GLI2) (GLI-A) in the cilium (Figure 9) [271,272]. In absence of the Hh ligand, GLI3is proteolytically cleaved to form a repressed form (GLI3R) [273,274]. Activation of Smo prevents this cleavage of GLI3 into GLI3R (Figure 9). The GLI proteins translocate to the nucleus and the balance of activators (GLI-A) to repressor (GLI3R) determine the expression of downstream target genes and the total output of the hedgehog pathway. In the absence of cilium, GLIA or GLI3R is not formed, resulting in an overall dysregulated pathway [273,274]. In addition to GLI proteins, several other hedgehog components, such as Suppressor of Fused (SuFu) and kinesins, acting similarly to Kif7 and Kif27, localise to the cilium [270]. Components of the IFT, BBSome, phospholipids (INPP5E affecting PI3K pathway) and Ca^+2^ flux in the cilium play a crucial role in the localisation of Hh signalling proteins in the cilium [275,276,277].

The hedgehog pathway is instrumental in renal development. Shh is the main ligand expressed in the distal embryonic epithelium of the ureter and medullary collecting ducts [278]. Mutations in Shh cause renal aplasia that affects nephron numbers during development [278,279]. Several other syndromes, such as Pallister–Hall syndrome, caused due to mutations in GLI3R, also result in renal patterning defects, emphasising the role of the hedgehog pathway in maintaining overall kidney structure and function [280,281].

Numerous studies document the dysregulation of the hedgehog pathway in renal cystic animal models and human patients. Components of hedgehog, including GLI2, were found to be upregulated in a genome-wide transcriptomic analysis of an ADPKD cohort [282]. Mutation in a transcription factor of GLI proteins, *Glis2* causes NPHP7 in humans and mice [49]. Glis2, in-vitro, downregulates the hedgehog pathway. The knockdown of *Glis2* also results in the transformation of renal epithelial cells into fibroblast-like appearance, implicating the role of the hedgehog pathway in maintenance of tubular cell differentiation state in kidneys [283]. Perinatal deletion of tetratricopeptide repeat-containing hedgehog modulator-1 [284] and conditional deletion of *IFT140* [285], both encoding components of IFT complex A, result in an increase in expression of *GLI* transcripts and cause cystic kidney diseases. Expression of *GLI* transcripts is also enhanced in *PKD1 cko* and Nek8 *jck* models [286]. The use of hedgehog inhibitors, such as cyclopamine, reduces or can even prevent cystic phenotypes in these models [286,287].

Despite this piece of evidence, the role of Hh signalling as a causal factor of PC1-mediated cystogenesis can be debated. Recently, Ma and colleagues showed that *PKD1* conditional mouse mutants presented cystic phenotype; albeit without a significant contribution from the Hh pathway [288]. The key difference in this study from other studies was the use of conditional genetic models. Activation or repression of Hh signalling did not affect the cystic phenotype in these mutants, again implying an accessory rather than a causal role of Hh in PKD.

### 6.7. Hippo Signalling

**Figure 10:**
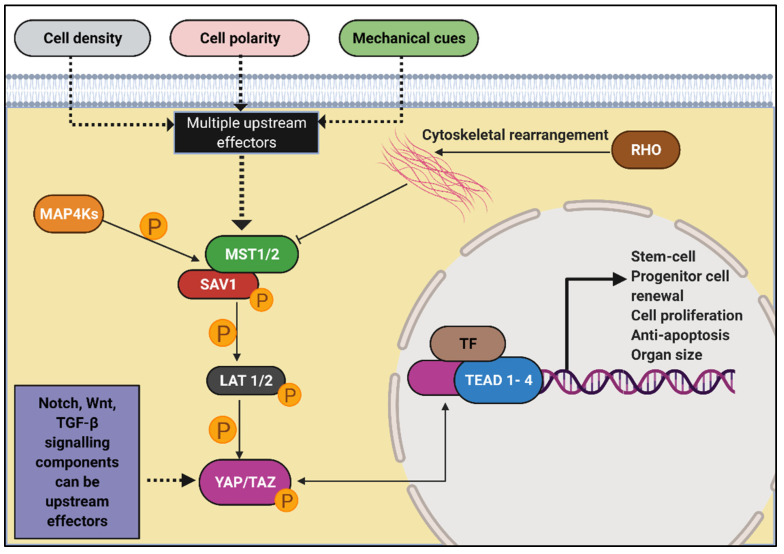
Key components of Hippo signalling upon activation: Various external stimuli (cell density, polarity, mechanical cues) and/or upstream cellular signals (mitogen-activated protein kinase (MAPK), RHO mediated signaling) can trigger the Hippo pathway. Upon activation, salvador family WW domain containing protein 1 (SAV1), macrophage stimulating 1/2 (MST1/2) interact to form a complex. This phosphorylates large neutral amino acids transporter small subunit 1/2 (LAT1/2) which in turn activates transcription co-activators yes-associated protein (YAP) and tafazzin (TAZ). Phosphorylated YAP/TAZ translocates to the nucleus and interacts with TEA domain transcription factor 1-4 (TEAD1-4) and other transcription factors to regulate the expression of Hippo pathway (Modified and redrawn using BioRender.com [290,291]).

The Hippo signalling pathway controls cell proliferation, stem cell renewal, apoptosis and is one of the central pathways responsible for organ size control. In brief, SAV1 and Mst1/2 kinases (ortholog of Drosophila Hippo) interact to form a complex (Figure 10). This complex activates LATS1/2 through phosphorylation, which in turn phosphorylates the two main downstream effects of the Hippo pathway, i.e., transcription co-activators YAP and TAZ. Phosphorylation of YAP and TAZ renders its inactivation and all downstream proteins in the cascade (Figure 10). Activated YAP and TAZ translocate into the nucleus and interact with TEAD1–4 along with other transcription factors. Each of these components has several upstream regulators modulating the output of Hippo. Overall, levels of nuclear YAP and TAZ regulate the expression of genes, promoting cell proliferation while inhibiting apoptosis (Figure 10) [289].

Considering the hyperproliferative nature of renal cells upon cystogenesis, Hippo is considered to have a role in cystic kidney disease [292,293]. However, recent reports have shown Hippo to be regulated in opposing fashion in NPHP as compared to ADPKD. This is interesting because of the contrasting phenotypic nature of the enlarged, extensively cystic ADPKD kidneys in comparison to the small, highly fibrotic kidney with fewer cysts in NPHP. The protein NPHP4 is an interactor and negative regulator of LATS [294]. Overexpression of NPHP4 results in elevated levels of activated YAP and TAZ [294]. NPHP9 and Nek8 missense and loss of function mutations differentially regulated YAP [295]. Overall, activation of Hippo signalling has been observed in NPHP. While this might seem counterintuitive to the observed NPHP phenotype, it is speculated that the reduced proliferative tone (relative to ADPKD) is what renders the Hippo pathway “on” in NPHP [292]. Conversely, in ADPKD, Hippo is observed to be “off”, presumably because of its over-proliferative nature. Cai et al. identified a RhoA–ROCK–Hippo–YAP/TAZ–c-Myc signalling axis downstream of *PKD1* via YAP phosphorylation [296]. The Sav1 mutant in the Hippo pathway shows tubular and glomerular cysts [297,298]. Taz itself is involved in the proteasomal degradation of PC2, however, a direct link connecting the Hippo pathway to cilia has not been found [299]. This pathway, therefore, requires more focused investigations on its ciliary links. The Hippo pathway is also considered difficult to target using YAP and TAZ inhibitors and activators, considering their role in oncogenesis [300].

### 6.8. DNA Damage Response (DDR)

**Figure 11:**
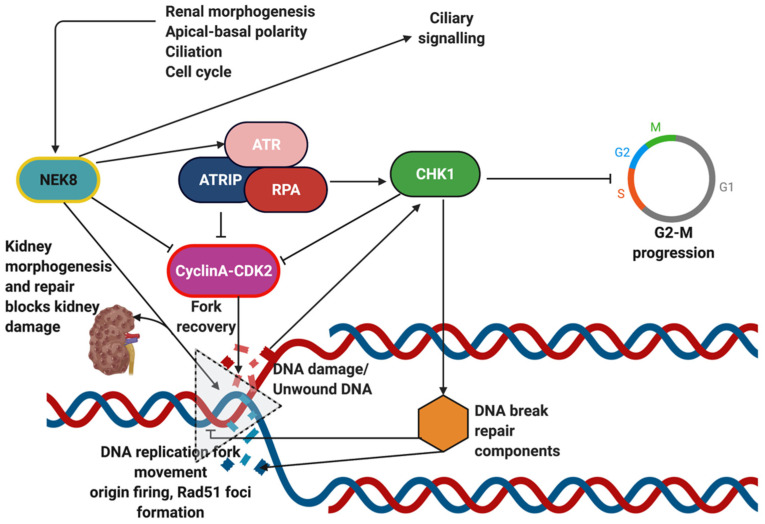
Role of NIMA related kinase 8 (Nek8) in the DNA Damage Response (DDR): ATR mediates one of the DDR pathways. Aberrations in ciliary protein Nek8 directly controls the activity of cyclin A kinase. Nek8 facilitates Rad51 foci formation and replication fork protection upon DNA damage, thereby protecting the kidney from DNA damage related injury and hypoxia. It also indirectly regulates the Ataxia Telangiectasia And Rad3-Related Protein (ATR)- Checkpoint Kinase 1 (Chk1) pathway, thus, having an overarching role in limiting DNA damage in response to replication stress (Modified and redrawn using BioRender.com [103,302]).

DDR is the collective term used for mechanisms that sense and repair mutagenic or pro-mutagenic lesions in DNA to maintain overall genome integrity [301]. The DDR broadly comprises of DNA damage sensors that recruit mediators to amplify damage signals to effector molecules. These effector molecules eventually regulate processes, such as cell cycle, chromatin remodelling, RNA processing, and apoptosis, etc., that act as a critical barrier against tumorigenesis. Although DDR via ATR-signalling has been implicated as a causal pathway in cystic kidney diseases, it might have a more intrinsic role in ciliary signalling, which will be described in this section (Figure 11).

Ataxia-telangiectasia-mutated (ATM), and ataxia telangiectasia and Rad3-related (ATR) are the two key DDR kinases activated by double-stranded breaks and replication protein A-coated ssDNA, respectively [303]. They target Checkpoint kinases 1 and 2, which eventually reduce CDK activities through a myriad of processes. CDK inhibition arrests cell cycle progression and prolongs the S-phase of the cell cycle to allow check-point proteins to repair damaged components, which, if not repaired, renders DDR in an activated state [303]. Chronic activation of the DDR triggers pro-apoptotic mechanisms to eliminate the possibility of tumorigenesis from the underlying damage [304]. Centrosomes, the microtubule organizing centre responsible for the organization of spindle fibres that facilitate chromosome segregation, have close associations to DDR components [305]. DNA damage causes aberrations in the architecture of the centrosome and pericentriolar material [306]. Abnormal ATR signalling is responsible for supernumerary centrosomes [307], that in turn results in defects in chromosome segregation. Supernumerary centrosomes sometimes nucleate more than one primary cilia, dysregulating the signalling cascades associated with it [308]. It has also been shown that the protein interaction modules in the centriolar satellites, along with centrosome proteins, enhance the CDK2 activity that regulates centriole replication [309]. Given such physical and functional links to centrosomes, DDR components are likely to be involved in ciliopathies.

A number of proteins, such as fanconi-associated nuclease 1 (FAN1), origin recognition complex subunit 1 (ORC1), envelope glycoprotein gp160 (VCP) and ATM interactor (ATMIN), are DDR proteins that have been implicated in ciliopathies [301]. Mutations in *MRE11* are known to cause NPHP, such as renal cysts [108]. SDCCAG8, also a centrosomal protein, is localised to nuclear foci upon DNA damage [310]. Mutations in this gene cause *NPHP10* through induction of replication stress. *Cep290* mutations have been implicated in NPHP6 and a range of other ciliopathies [89]. This protein is also centrosomal and regulates replication stress. Cep164 localised in centrosomes is regulated by DDR kinases. It promotes DNA damage repair responses and, when mutated, causes NPHP15. Conversely, many ciliary proteins are now being investigated for their roles in the DDR. Nek8 causing NPHP9 is a protein that not only localises to the inversin compartment in the cilia but also associates with the centrosome and nucleus to interact with ATR, CHK1 and ATRIP, to regulate the CDK2 activity mediating the DDR (Figure 11) [103]. Nek8 regulates Rad51 foci formation and replication fork protection upon DNA damage [102]. Missense mutations in Nek8 thereby result in increased γH2AX foci (phosphorylated form of histone variant H2AX at Ser-139) in the kidney, characteristic of defects in DDR via ATR signalling [103,311].

A study by Stiff et al. proposed that ATR could be activated in the absence of DNA damage, in a cilia dependent mechanism, possibly through mechanotransduction [312]. In another study by Kumar et al., mechanical stress was shown to activate ATR at nuclear envelopes [313]. Therefore, ciliary proteins with roles in DDR can be speculated to have dual or multiple overlaps in signalling pathways involving mechanotransduction or other biochemical pathways, or a combination thereof, thus emphasising their causal role in renal cystogenesis beyond the classical DDR.

### 6.9. Cilia Dependent Cyst Activation (CDCA)

The existence of CDCA was proposed by Ma et al. in 2017 [314]. This was based on a review of the effect of cilia (not ciliary proteins) on cystogenesis that stemmed from a series of experiments performed by Ma and colleagues in Stefan Somlo’s laboratory in USA. As is clear from the previous sections, loss of function of several ciliary proteins leads to the formation of renal cysts. Hence it might seem intuitive to think that removing cilia from renal cells would aggravate cystogenic processes. However, the ablation of cilia in early and adult activated murine models of PC1, as well as PC2, helped suppress cystogenesis [315]. Furthermore, transgenic overexpression of PC1 in mutant cells devoid of cilia did not alleviate or worsen the cystogenic burden [315]. Interestingly, the severity of disease correlated to the period of disappearance of polycystins followed by cilia removal in double knockouts [315]. This PC independent, cilia dependent mechanism governing cystogenesis and disease severity was termed as CDCA. Ma et al. proposed that the PC1/PC2-CDCA axis, under normal conditions, exists in dynamic balance to regulate renal tubule diameter [314]. However, upon modulation from external cues, such as luminal flow or ligand binding (e.g., Wnts), PCs act as the rate-limiting component for CDCA, such that chronic PC dosage below a certain threshold activates this otherwise indolent pathway. They propose that upon activation, components of CDCA stimulate the remodelling of the basement membrane of tubules and the overall cell proliferation that leads to cystogenesis. Therefore, the ablation of cilia at this point substantially reduces this cilium dependent pathogenesis, regardless of PC function [315].

This theory, while relevant, requires experimental evidence to shed light on the underlying mechanism of PCs activating this otherwise inert process. Moreover, once activated, the irreversible nature of CDCA, despite transgenic overexpression of functional PCs, requires research; especially if the PC threshold were to be the only triggers activating this pathway. To reach that point, identification of at least a few key components of the CDCA would be necessary to perform conditional genetic experiments on cells with fully formed cilia, to shed light on the relation of PCs with CDCA. Drivers of CDCA can be speculated to belong to any of the niche ciliary signalling pathways, or could be components with multifaceted roles and links to cilia (e.g., DDR proteins). Elucidation of this process is likely to be key to linking other related cystogenic pathologies and determining the intrinsic ciliary pathways involved in cystogenesis, which is otherwise overshadowed by the role of polycystins in isolation.

## 7. Diagnosis, Screening and Prevention

NPHP diagnosis is mostly clinical, supported by ultrasonographic and histopathological features in conjunction with extrarenal features. The first symptoms could be noticed antenatally or in early infancy, but the median age is 6 years. At that stage, there is marked polyuria and polydypsia (especially nocturnal), decreased ability to concentrate urine and, later, growth retardation. Blood pressure remains within normal limits and there is no oedema, proteinuria or haematuria [316]. Creatinine rises on an average by age nine [13], and CKD subsequently leads to anaemia and uraemia with nausea and weakness. Ultrasonographically, there is increased echogenicity due to cysts and disruption of corticomedullary differentiation, where kidneys could be big or small [17]. On histopathological analysis, cysts and interstitial fibrosis, with disintegration of the basement membrane in the tubules, are observed [25].

There may be significant variability of presentation within families [317], and 10–20% of renal cases will be accompanied by involvement of other organs, mostly eyes, brain, liver and skeletal system [18]. Clinical suspicion for NPHP should be high in patients with:

(1) Positive family history of either cystic kidneys, CKD, need for renal replacement therapy or renal transplant, or consanguineous families;
(2) Childhood onset CKD, often associated with hyperechoic kidneys prenatally;
(3) Clinically concentration defect, polyuria, polydipsia, and hyponatremia;
(4) Ultrasound findings confirming bilateral cystic kidneys;
(5) Associated syndromic features, especially if involving eyes (e.g., retinitis pigmentosa); brain (e.g., in Joubert syndrome), musculoskeletal system (as in BBS), developmental delays, autism, cardiac features (especially situs inversus), and short stature etc.

Genetic diagnosis of NPHP is now possible in patients with disease suspected clinically, but remains complex and resource consuming. As detailed earlier, only about 30% of patients will have a clear genetic mutation identified [55]; NPHP1 mutation is most frequently detected (25–62% of genetic testing [318]). Advances in genetic screening have allowed for more complex testing, including NGS (whole genome or exome) [319], and are able to diagnose about 20% of causes of genetic renal diseases in adults [320], but recent studies revealed that, in children, whole exome sequencing could provide a genetic diagnosis in 46–63% of cases [321,322]. If diagnosis is confirmed, this can facilitate prognosis and further management, without additional diagnostic or surveillance imaging, but needs to be supported by genetic counselling. Access to and cost of genetic testing is variable, in some countries being available only in research units [323], and often with significant turnaround time. Despite this, implementation of testing and registries has allowed the creation of comprehensive databases of genotype-phenotype correlation, which will help with future diagnosis [324]. Registries exist in the USA [56], China [325], Germany, Austria, and Denmark [318], and Holland [326].

Contrary to ADPKD, where a clearly identified diagnostic process exists [327], there is no strict diagnostic criteria for NPHP, unless genetic diagnosis is made. The majority of diagnoses are made postnatally, with only a small percentage of children suspected to have NPHP following antenatal diagnosis. In families with a known mutation, diagnosis is simpler via genetic testing of the affected child or foetus. There is no prevention, and paediatric care focuses on prevention of morbidity and slowing disease progression.

## 8. Management

While Tolvaptan, an arginine vasopressin (AVP) V2 receptor antagonist, has been shown to slow the progression of disease and cysts growth in ADPKD [328], other medications targeting upregulated pathways were not as successful, even though studies in NPHP animal models were promising. Treatment for NPHP remains mostly supportive. Therapy focuses on normalisation of electrolytes, salt substitution, hypertension management improvement of proteinuria and CKD management.

Syndromic patients with multiple extrarenal features benefit from multidisciplinary team reviews [329], with involvement of an ophthalmologist, developmental paediatrician, neurologist, physiotherapist, orthopaedic surgeon, geneticist, genetic counsellor, endocrinologist, physiotherapist, dietician and others. If families are referred to a genetic service, prospective prenatal testing could be appropriate for any subsequent pregnancies.

Prevention of complications include immunisations as per local schedules, inclusive of annual influenza vaccines, optimization of nutrition and growth, with involvement of a dietician, an endocrinologist, or indeed both, where required, and avoidance of nephrotoxins. Follow up depends on the condition identified, staging of CKD and presence or absence of associated features, but focuses on optimisation of kidney function, growth and development, blood pressure management if required and prevention of complications, and is tailored to each patient, given intragenomic and even intrafamilial variability.

Outcomes in NPHP are variable, depend on the mutation, stage of CKD on presentation and presence of extrarenal features. Some phenotypes may be severe enough to be lethal (e.g., Jeune Dystrophy [330] or MKS [331]), while juvenile NPHP patients may only suffer mild kidney impairment with slow progression. ESRD and the need for renal replacement therapy (RRT) may be evident in either early infancy or in later adulthood. Following renal transplantation, NPHP patients have an excellent prognosis, with better creatinine clearance and slower decline in creatinine when compared to graft recipients with other diagnoses [332], and higher rates of graft survival at 5 and 10 years (both 95.5%) when compared to non NPHP patients (94.7% and 86.8%, respectively) [333]. Future directions may focus on gene therapy in some selected conditions, with initial promising results with antisense oligonucleotides in NPHP6 mutation [334,335] in Leber’s amaurosis, but given the low numbers of patients and lack of randomized clinical trials, this is unlikely to happen in the near future.

## 9. Quality of Life

There are no large studies examining quality of life in patients with NPHP. Common complaints assessed in registries are fatigue, musculoskeletal pains, and symptoms of uraemia in patients with advanced CKD [326].

## 10. Outlook

Over the last two decades, the molecular nephrology community has taken huge strides in the understanding of pathways driving cystic kidney diseases. Intricately designed experiments alongside robust discovery-based workflows using various mutant models have yielded valuable insights that establish how primary cilia dysfunction results in the modulation of cellular pathways that drive cystogenesis [60,314]. This includes the identification of cilia centric hedgehog, mTOR, canonical and non-canonical Wnt, PI3K-Akt and other signalling pathways as being altered in cystogenesis. However, recent studies have established that while these pathways may be dysregulated in PKD, they may not directly affect the process of cystogenesis [264,288].

Most molecular studies on cystogenesis have focused on ADPKD causing mutations in *Pkd1* and *Pkd2* genes (encoding PC1 and PC2, respectively) due to their epidemiological burden in comparison with other genes associated with renal cysts. The irreversible nature of CDCA described by Ma et al. [314] as being responsible for cystogenesis despite PC1 overexpression, however, needs dedicated investigation [314,315]. This is important to address because a threshold of functional polycystins is proposed to be the sole upstream trigger for CDCA activation. While studies on PC1 and PC2 mutations have been invaluable in establishing our current understanding of cystic kidney diseases, it is well established that cystogenic pathways can be triggered by mutations in a vast set of cystogenes [3]. Given the “cilia-dependent” nature of CDCA, the dependence on the optimal functioning of all ciliary proteins as critical mediators of the PC1 homeostatic signal is implied [315]. These ciliary proteins can be conceptualised to be a sphere of interconnecting nodes, whose primary role in the kidney could be imparting polarity through the developmental stages and controlling cell proliferation after terminal differentiation, thereby maintaining tubule diameter and cell morphology [314]. To determine the underlying mechanisms driving cystogenesis, there is a necessity to study cystogenes besides PC1 and PC2 and to consider their established roles more holistically rather than in isolation, irrespective of epidemiological burden. This is where NPHP proteins can be investigated to not only better understand the molecular process of renal cystogenesis but also to obtain insights on the role of ciliary proteins in multiple organs.

With recent evidence of certain ciliary proteins localising in sites other than the primary cilia, there is a consensus building towards exploring extra-ciliary roles of ciliary proteins [336,337]. With kidney organoid development, next-generation sequencing and single cell technologies becoming readily accessible, there remains huge scope for researchers to identify novel NPHP causing genes, either through genetic screens in clinics or mutagenesis screens coupled with 3D culturing. Identification of causal proteins would bring us closer to a holistic understanding of the biomolecular steps involved in cyst formation, factoring in as a vital resource towards the development of novel and effective disease management strategies for cystic pathologies, be it NPHP or other diseases under the PKD umbrella.

## Figures and Tables

**Table 1:** Monogenic genes causing NPHP and their phenotypic spectrum from associated ciliopathies.

Gene	Chromosome	Protein	Location	Mode of Inheritance	Animal Model	Protein Families	Human Disease Phenotype *	
							Kidney	Extra Renal Presentations/Syndromes
*NPHP1*	2q12.3	Nephrocystin-1	Adherens junctions, focal adhesion, transition zone	AR	Mice: Nphp1 del20/del20; Zebrafish: MO, morpholino antisense oligonucleotides	Nephrocystin-1; Domain: coiled-coil, SH3 domain	NPHP juvenile	Eye (retinitis pigmentosa, oculomotor apraxia), brain (cerebellar vermis hypoplasia)
*NPHP2/INVS*	9q31.1	Inversin	Inversin compartment	AR	Mice: Inv -/-, mice, Zebrafish: MO, morpholino antisense oligonucleotides	Domain: ANK repeat, Repeat	NPHP infantile	Eye (retinitis pigmentosa), liver (bile duct proliferation, liver fibrosis), laterality defects (Situs inversus), pulmonary hypoplasia, oligohydramnios
*NPHP3*	3q22.1	Nephrocystin-3	Inversin compartment, axoneme	AR	Mice: pcy, Nphp-/- Zebrafish: MO, morpholino antisense oligonucleotides	Coiled-coil, Repeat, TPR repeat	NPHP (including infantile)	Eye (retinitis pigmentosa), liver (liver fibrosis, liver cysts), laterality defects (Situs inversus), heart (congenital heart disease), multiple organ polycystic disease, Meckel-–Gruber syndrome
*NPHP4*	1p36.31	Nephrocystin-4	Transition zone	AR	Mice: Nphp4em1(IMPC)Bay; Zebrafish: MO, morpholino antisense oligonucleotides	NPHP4 Family	NPHP	Eye (retinitis pigmentosa, oculomotor apraxia), liver (bile duct proliferation, liver fibrosis), laterality defects (heterotaxia), heart (congenital heart disease)
*NPHP5/IQCB1*	3q13.33	Nephrocystin-5/IQ motif containing B1	Transition zone, basal body	AR	Mice: Iqcb1em1(IMPC)Bay; Zebrafish: MO, morpholino antisense oligonucleotides	Coiled-coil, Repeat	NPHP	Eye (retinitis pigmentosa)
*NPHP6/CEP290*	12q21.32	Nephrocystin-6/centrosomal protein 290	Transition zone, centrosome	AR	Mice: Cep290em1(IMPC) Mbp; rd16; Zebrafish: MO, morpholino antisense oligonucleotides-	Coiled-coil	NPHP	Eye (retinitis pigmentosa), brain (cerebellar vermis hypoplasia, congenital brain defect), liver (bile duct proliferation, liver fibrosis), skeletal defects (polydactyly), heart (ventricular septal defect), Meckel–Gruber syndrome, Bardet–Biedl syndrome
*NPHP7/GLIS2*	16p13.3	Nephrocystin-7/GLI similar 2	Nucleus	AR	Mice:Glis2tm1Tre/Glis2tm1Tre; Zebrafish: MO, morpholino antisense oligonucleotides	GLI C2H2-type zinc-finger protein family.	NPHP	
*NPHP8/RPGRIP1L/MKS5*	16q12.2	Nephrocystin-8/RPGRIP1-like	Transition zone	AR	Mice: Rpgrip1l -/- embryos; Ftm	Coiled-coil, Signal	NPHP	Eye (retinitis pigmentosa, oculomotor apraxia), brain (cerebellar vermis hypoplasia, congenital brain defect), liver (bile duct proliferation, liver fibrosis), skeletal defects (polydactyly), Meckel–Gruber syndrome, Bardet–Biedl syndrome
*NPHP9/NEK8*	17q11.2	Nephrocystin-9/NIMA-related kinase 8	Inversin compartment	AR	Mice: Nek8 jck; Nek8-Zebrafish: MO, atg morpholino (5′-TTCTCATACTTCTCCATGTTTTCGC-3′); Rat: LPK	Protein kinase superfamily. NEK Ser/Thr protein kinase family. NIMA subfamily	NPHP (including infantile)	Liver (liver fibrosis), heart (congenital heart disease), Meckel–Gruber syndrome
*NPHP10/SDCCAG8/SLSN7*	1q43-q44	Nephrocystin-10/Serologically defined colon cancer antigen 8	Basal body	AR	Mice: Sdccag8tm1a(EUCOMM)Wtsi; Zebrafish: MO, morpholino antisense oligonucleotides	Domain: coiled-coil	NPHP	Eye (retinitis pigmentosa), brain (intellectual disability), Bardet–Biedl syndrome
*NPHP11/TMEM67/MKS3*	8q22.1	Nephrocystin-11/Transmembrane protein 67	Transition zone	AR		Domain: Signal, Transmembrane, Transmembrane helix	NPHP	Eye (retinitis pigmentosa, oculomotor apraxia, coloboma), brain (cerebellar vermis hypoplasia), liver (bile duct proliferation, liver fibrosis), skeletal defects (polydactyly), Meckel–Gruber syndrome
*NPHP12/TTC21B/JBTS11*	2q24.3	Nephrocystin-12/Intraflagellar transport protein	IFT-A	AR		TTC21 family; Domain: Repeat, TPR repeat	NPHP	Brain (cerebellar vermis hypoplasia), skeletal defects (Jeune asphyxiating thoracic dystrophy), Meckel–Gruber syndrome
*NPHP13/WDR19*	4p14	Nephrocystin-13/WD repeat domain 19/IFT protein 144	IFT-A	AR		Domain: Repeat, TPR repeat, WD repeat	NPHP	Eye (retinitis pigmentosa), liver (liver fibrosis), skeletal defects (Jeune asphyxiating thoracic dystrophy, cranioectodermal dysplasia)
*NPHP14/ZNF423*	16q12.1	Nephrocystin-14/Zinc finger protein 423	Nucleus	AR/AD		Krueppel C2H2-type zinc-finger protein family; Domain: Repeat, Zinc-finger	NPHP	Eye (retinitis pigmentosa), brain (cerebellar vermis hypoplasia), laterality defects (situs inversus)
*NPHP15/CEP164*	11q23.3	Nephrocystin-15 centrosomal protein 164	Basal body	AR		Domain: Coiled-coil	NPHP	Eye (retinitis pigmentosa), brain (cerebellar vermis hypoplasia, intellectual disability), liver (liver fibrosis), skeletal defects (polydactyly), obesity
*NPHP16/ANKS6*	9q22.33	Nephrocystin-16/ANKS6	Axoneme, inversion compartment	AR	Mice: Anks6tm1b(KOMP)Wtsi; Zebrafish: MO, antisense morpholinos	Domain: Ankyrin repeat, Repeat	NPHP	Liver (liver fibrosis), laterality defects (situs inversus), heart (congenital heart disease)
*NPHP17/IFT172*	2p23.3	Nephrocystin-17/IFT protein 172	IFT-B	AR		IFT172 family- Domain: Repeat, TPR repeat, WD repeat	NPHP	Eye (retinitis pigmentosa), brain (cerebellar vermis hypoplasia, intellectual disability), liver (liver fibrosis), skeletal defects (short-rib thoracic dysplasia, polydactyly), ventricular septal defect, obesity
*NPHP18/CEP83*	12q22	Nephrocystin-18/centrosomal protein 83	Basal body	AR		CEP83 family; Domain: coiled-coil	NPHP (including infantile)	Eye (retinitis pigmentosa), brain (hydrocephalus, intellectual disability), liver (liver fibrosis)
*NPHP19/DCDC2*	6p22.3	Doublecortin domain-containing protein 2	Axoneme	AR		Domain: Repeat	NPHP	Liver (liver fibrosis)
*NPHP20/MAPKBP1*	15q15.1	Mitogen-activated protein kinase binding protein 1	Cytoplasm	AR		Domain: Repeat, WD repeat	NPHP	
*NPHP1L/XPNPEP3*	22q13	X-prolyl aminopeptidase 3	Mitochondria	AR		Peptidase M24B family; Domain: Transit peptide	NPHP, gout	Neurological disorder (essential tremor), ear (high frequency sensorineural hearing loss), brain (arachnoid cysts)
*NPHP2L/SLC41A1*	1q32.1	Solute carrier family 41-member 1	Tubules at the borders of the cortex and medulla	AR		SLC41A transporter family; Domain: Repeat, Transmembrane, Transmembrane helix	NPHP	Skeletal defects (ventral body curvature), brain (hydrocephalus)
*TRAF3IP1*	2q37.3	TRAF3 interacting protein 1	Axonemes, basal bodies	AR	Mice: Traf3ip1em1(IMPC)Bay	TRAF3IP1 family; Domain: coiled-coil	NPHP	Eye (retinitis pigmentosa), brain (intellectual disability), skeletal anomaly (brachydactyly)
*AH11/JBTS3*	6q23.3	Jouberin	Basal bodies	AR	Mice:Ahi1tm1Jgg/Ahi1tm1Jgg	Domain: coiled-coil, Repeat, SH3 domain, WD repeat	NPHP, rare	Eye (retinitis pigmentosa, oculomotor apraxia), brain (cerebellar vermis hypoplasia, intellectual disability)
*JBTS9/CC2D2A/MKS6*	4p15.32	Coiled-coil and C2 domain containing 2A	Basal bodies	AR	Mice: Cc2d2atm1a(EUCOMM)Wtsi	Domain: coiled-coil	NPHP	Eye (retinitis pigmentosa, oculomotor apraxia), brain (cerebellar vermis hypoplasia, intellectual disability), liver (liver fibrosis), Meckel–Gruber syndrome
*FAN1 KIAA1018, MTMR15*	15q13.3	Fanconi-associated nuclease 1	nucleus	AR	Mice: Fan1(nd/nd) Zebrafish: MO, antisense morpholinos	Domain: coiled-coil, D-box, KEN box	NPHP, Interstitial nephritis, karyomegalic	

Sourced from ONIM [124] and Uniprot [125]: * Originally compiled by Braun and Hildebrandt, 2017, ©Cold Spring Harbor Laboratory Press [3]; adapted and updated. Original citations for individual NPHP proteins in Section 5.

## Data Availability

Not applicable.
